# Pancreatitis in Patients Receiving Tirzepatide (Mounjaro): A One-Year Audit in a UK District General Hospital

**DOI:** 10.7759/cureus.96974

**Published:** 2025-11-16

**Authors:** Asmita Hossain, Muhammad Fahim, Moumn Abdalla, Khaldoun Fozo, Minthu Mon, Botond Kaholics, Safana Veettil, Ajay Belgaumkar, Kirstin Carswell

**Affiliations:** 1 Surgery, Surrey and Sussex Healthcare NHS Trust, Redhill, GBR; 2 General and Colorectal Surgery, Surrey and Sussex Healthcare NHS Trust, Redhill, GBR; 3 Urology, Surrey and Sussex Healthcare NHS Trust, Redhill, GBR; 4 Breast Surgery, Surrey and Sussex Healthcare NHS Trust, Redhill, GBR; 5 Upper GI Surgery, Surrey and Sussex Healthcare NHS Trust, Redhill, GBR

**Keywords:** bariatric medicine, gallstone pancreatitis, obesity treatment, pancreatitis causes, tirzepatide or mounjaro

## Abstract

Background: Tirzepatide (Mounjaro) is a dual glucose-dependent insulinotropic polypeptide (GIP) and glucagon-like peptide-1 (GLP-1) receptor agonist increasingly prescribed for type 2 diabetes and obesity due to its efficacy in weight loss and glycaemic control. In US FDA-reviewed clinical trials, pancreatitis occurred rarely, at rates of approximately 0.32-0.39% across all doses, comparable to placebo groups. Nevertheless, concerns remain about potential drug-associated pancreatitis, with case reports describing variable severity.

Aim: To determine the frequency and clinical characteristics of pancreatitis among patients receiving tirzepatide in a UK District General Hospital over a 12-month period.

Methods: This retrospective single-centre audit reviewed 222 inpatient admissions coded as acute or chronic pancreatitis between February 2024 and February 2025. Demographics, comorbidities, drug history, imaging, and outcomes were extracted from electronic health records, focusing on anti-diabetic and anti-obesity medications. Acute and chronic cases were analysed separately to maintain the cohort context.

Results: Of 222 patients, four (1.8%) were prescribed tirzepatide at admission. All were female with a body mass index (BMI) > 24 kg/m² (overweight to obese range), presenting with first-episode acute pancreatitis. All cases were mild, with no intensive care admission, necrosis, or pseudocyst formation. Confounding risk factors were frequent: two had gallstones, one reported alcohol intake, and one had no alternative cause identified. These findings align with the literature suggesting that rapid weight loss may increase gallstone-related pancreatitis risk.

Conclusion: In this single-centre audit, pancreatitis in patients receiving tirzepatide was rare, mild, and commonly associated with other aetiologies, such as gallstones or alcohol. No cases of chronic pancreatitis occurred among tirzepatide users. Although causality cannot be established, the overall risk appears low. Clinicians should remain vigilant during early treatment phases when weight loss is greatest. Further multicentre studies with prescribing denominator data are warranted to clarify incidence and potential preventive measures, such as short-term ursodeoxycholic acid prophylaxis in high-risk individuals.

## Introduction

Tirzepatide (Mounjaro) is a novel dual agonist of glucose-dependent insulinotropic polypeptide (GIP) and glucagon-like peptide-1 (GLP-1) receptors, approved in 2022 for the management of type 2 diabetes mellitus (T2DM) and more recently licensed for obesity due to its notable effects on glycaemic control and weight loss [1].

Despite these metabolic benefits, concerns regarding potential drug-induced pancreatitis have persisted. In US Food and Drug Administration (FDA)-reviewed clinical trials of tirzepatide, pancreatitis occurred rarely at rates of approximately 0.32-0.39% across all doses, comparable to rates observed in placebo-treated participants. The FDA prescribing information for tirzepatide, therefore, includes a warning for acute pancreatitis, reflecting class-related concerns previously observed with GLP-1 receptor agonists [2].

While early post-marketing reports suggested an increased risk of pancreatitis with incretin-based therapies, subsequent large-scale meta-analyses have not shown a statistically significant increase in pancreatitis incidence among GLP-1 receptor agonist users compared with other anti-diabetic agents [3-5]. The most recent pooled analyses further confirmed that the absolute risk of pancreatitis with GLP-1 receptor agonists, including tirzepatide, remains below 1%, with no clear causal relationship established [4,5]. Furthermore, the physiological actions of incretin-based agents, such as enhanced insulin secretion and delayed gastric emptying, have been well characterised without consistent evidence of direct pancreatic injury [6].

Nevertheless, a small number of isolated case reports of acute and necrotising pancreatitis associated with tirzepatide have been published in clinical journals and pharmacovigilance sources, indicating that, although rare, the possibility of drug-related pancreatitis cannot be excluded entirely [7,8]. Additionally, rapid weight loss associated with tirzepatide therapy may theoretically promote gallstone formation through gallbladder hypomotility and bile stasis, which are recognised causes of secondary pancreatitis [9].

Given tirzepatide’s expanding clinical use across diabetes and obesity populations in the UK [1], real-world evidence assessing its pancreatic safety remains limited. Therefore, this retrospective audit aimed to evaluate the association between tirzepatide use and pancreatitis, both acute and chronic, over a 12-month period in a UK District General Hospital (DGH).

## Materials and methods

Study design and setting

This was a retrospective, single-centre audit conducted at a UK DGH, reviewing all inpatient admissions coded as acute or chronic pancreatitis between February 2024 and February 2025. A total of 222 patients were identified using standard hospital diagnostic coding (ICD-10 system, routinely applied in NHS secondary care) and verified through electronic medical records. Coding accuracy was checked by reviewing discharge summaries.

Data collection

Data were extracted from the hospital’s electronic health record system using a standardised proforma developed by the audit team. Information collected included patient demographics (age, sex, body mass index), comorbidities, relevant past medical history, drug history, radiological findings, biochemical results, and clinical outcomes.

Particular attention was given to patients prescribed anti-diabetic or anti-obesity medications, especially tirzepatide (Mounjaro), to evaluate possible associations with acute or chronic pancreatitis. Diagnosis and disease severity were confirmed based on documented clinical assessment, biochemical results (elevated serum amylase or lipase), and radiological findings recorded in the patient notes, in line with the Revised Atlanta Classification. The Ranson criteria were also applied on admission, where applicable and where sufficient data were available.

Data analysis

Data were organised and analysed descriptively using Microsoft Excel (Microsoft Corp., Redmond, WA). Continuous variables were summarised as means or medians, and categorical variables as frequencies and percentages. As only four patients were taking tirzepatide, the analysis was exploratory, and no formal statistical testing was performed.

Ethical considerations

This audit was registered with the local Surgical Audit Team at Surrey and Sussex Healthcare NHS Trust (Audit ID: 3205) and presented at the departmental general surgical audit meeting. The project was conducted in accordance with institutional audit policies and GDPR data protection standards. As it involved anonymised retrospective data review without direct patient contact, formal research ethics approval was not required under NHS Health Research Authority guidance.

## Results

Demographics 

A total of 222 unique patients were admitted with pancreatitis during the 12-month study period. The mean age was 57 years, and 122 (55%) were male and 100 (45%) were female, giving a male-to-female ratio of 1.22:1. The mean body mass index (BMI) was 30.9 kg/m². The mean length of hospital stay (LOS) was 5.7 days (median: 3.9, range: 0.26-45.1).

Comorbidities 

Among 138 patients with documented comorbidity data, the most frequent conditions were obesity/high BMI in 34 (24.6%), T2DM in 23 (16.7%), gallstones in 27 (19.6%), and alcohol use in 27 (19.6%), with nine patients (6.5%) having both obesity and T2DM. These represent key predisposing factors within the pancreatitis cohort. Comorbidity data were unavailable for 84 patients due to incomplete documentation in electronic records.

Associated aetiologies

Across all 222 admissions, identifiable precipitating factors for pancreatitis included gallstones in 64 patients (28.8%), alcohol use in 59 (26.6%), obesity or high BMI in 44 (19.8%), and T2DM in 33 (14.9%). A smaller subset was attributed to post-endoscopic retrograde cholangiopancreatography (ERCP) pancreatitis (six cases, 2.7%) and idiopathic pancreatitis (five cases, 2.3%). Rare causes included groove pancreatitis, pancreatic divisum, post-gastric sleeve pancreatitis, and pancreatic or duodenal adenocarcinoma.

**Figure 1:**
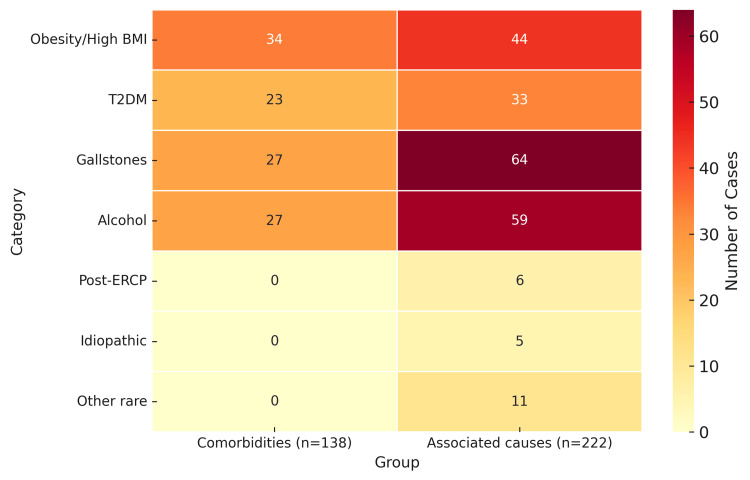
Comparison of comorbidities (n = 138) and associated aetiologies (n = 222) of pancreatitis Heatmap showing the distribution of comorbidities and associated causes of pancreatitis Abbreviations: BMI – body mass index; T2DM – type 2 diabetes mellitus; ERCP - endoscopic retrograde cholangiopancreatography

The comparative distribution of comorbidities and associated causes of pancreatitis is illustrated in Figure 1.

Tirzepatide (Mounjaro)-exposed cases 

Four patients (1.8% of total admissions) were prescribed tirzepatide at the time of admission. All were female with BMI > 24 kg/m² (overweight to obese range) and presented with first-episode acute pancreatitis. Two cases were gallstone-related, one had alcohol exposure, and one had no alternative cause identified radiologically. All four experienced mild disease, with short hospital stays and no intensive care admission, necrosis, or pseudocyst formation.

Among patients with a confirmed classification, 195 (87.4%) presented with acute pancreatitis, and 28 (12.6%) had chronic pancreatitis, indicating that most admissions were new, acute presentations.

Sequelae 

Follow-up data were available for 168 patients. Among these, one patient (0.6%) died, 10 (6.0%) developed chronic pancreatitis, four (2.4%) developed necrosis, four (2.4%) formed pseudocysts, two (1.2%) had abscess or peripancreatic collections, and three (1.8%) developed common bile duct (CBD) dilatation. Follow-up included both in-hospital outcomes and post-discharge clinic review, where available.

**Figure 2:**
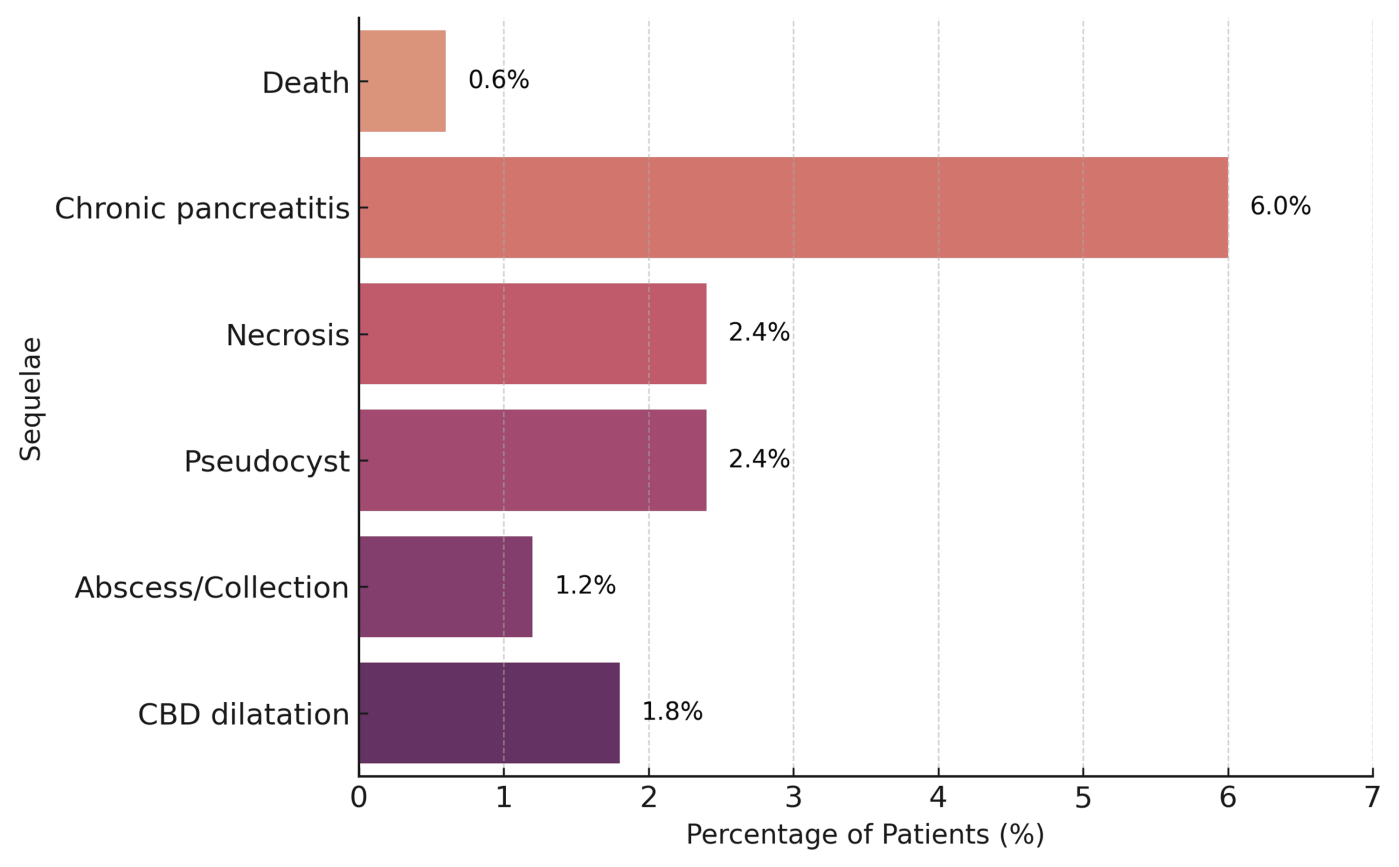
Sequelae and complications following pancreatitis (n = 168) Abbreviations: CBD - common bile duct

Overall, approximately 14-15% of patients experienced a clinically significant complication, defined as events requiring intervention, prolonged hospital stay, or intensive care admission. The distribution of sequelae is illustrated in Figure 2.

Computed tomography (CT) scan and ICU findings 

Among 222 patients with imaging and admission data, seven (3.2%) required ICU admission. A CT scan was performed in 156 (71.2%) patients. Fat stranding, consistent with mild pancreatitis, was observed in 54 (24.7%) cases. Pseudocysts and necrosis were each reported in 10 (4.6%) patients, indicating that severe pancreatitis (radiologically defined by necrosis or pseudocyst formation) was uncommon.

## Discussion

This one-year audit of 222 pancreatitis admissions in a UK DGH identified four patients (1.8%) who developed acute pancreatitis while receiving tirzepatide (Mounjaro). All cases were mild, with no necrosis, pseudocyst formation, or requirement for intensive care. Three of the four patients had confounding risk factors, namely, gallstones or alcohol use, consistent with published evidence that most GLP-1 and GIP receptor agonist-associated pancreatitis events occur in the presence of traditional aetiologies [3,5]. Disease severity in all cases was classified according to the Revised Atlanta criteria.

Our findings align with international evidence indicating that the absolute risk of pancreatitis among patients treated with tirzepatide or other GLP-1 receptor agonists is very low (<1%) and comparable to that of other antidiabetic therapies [3-5]. For example, Kristensen et al. [3] reported no significant increase in pancreatitis across major GLP-1 receptor agonist trials, while Zeng et al. [4] found pooled incidence rates below 1% with no established causal link. Meta-analyses of randomised controlled trials have demonstrated that pancreatitis events during tirzepatide therapy are rare and not statistically different from rates observed with placebo or insulin comparators [4,5]. While isolated case reports of acute and necrotising pancreatitis have been described, these remain exceptional occurrences in real-world practice [7,8].

The proposed pathophysiological mechanisms linking tirzepatide to pancreatitis appear indirect and multifactorial. Dual GIP/GLP-1 receptor agonism enhances insulin secretion, suppresses glucagon, and delays gastric emptying, which can contribute to rapid weight loss [1,6]. This weight reduction may, in turn, cause gallbladder hypomotility and bile stasis, predisposing to gallstone formation, a well-established cause of secondary acute pancreatitis [9]. Additional hypotheses include transient increases in pancreatic ductal pressure, local enzyme activation, and inflammatory cytokine modulation, though these lack definitive mechanistic evidence [6,9].

Rapid weight loss, whether due to tirzepatide therapy or bariatric surgery, has long been recognised to increase the risk of gallstone formation and gallstone-related pancreatitis [9]. Randomised trials in bariatric cohorts have shown that ursodeoxycholic acid (UDCA) prophylaxis significantly reduces gallstone formation and biliary complications. By analogy, early UDCA prophylaxis could be considered for high-risk tirzepatide users, although specific data in GLP-1/GIP-treated populations are currently lacking [9].

In June 2025, the UK Medicines and Healthcare products Regulatory Agency (MHRA) and Genomics England launched the Yellow Card Biobank initiative to investigate genetic susceptibility to rare adverse drug reactions, including incretin-related pancreatitis. This reflects continued pharmacovigilance interest in the safety of GLP-1 receptor agonists despite their overall favourable benefit-risk profile.

This audit has several important limitations. It was a single-centre, single-cycle retrospective study conducted in a district general hospital, limiting external validity. The small number of tirzepatide-associated cases (n = 4) precluded meaningful statistical analysis. Data on tirzepatide use were based solely on recorded clinical history, and many patients may have obtained the drug privately or via general practice, resulting in incomplete capture of exposure data. The absence of pharmacy prescribing denominators further prevented estimation of true incidence or relative risk compared with unexposed cohorts.

Additionally, no formal statistical testing was performed, and the analysis relied on descriptive trends rather than inferential comparisons. The retrospective design introduces potential for coding inaccuracies, missing documentation, and underreporting of confounding risk factors such as alcohol or gallstones, which may have influenced case classification.

Further prospective, multicentre studies integrating hospital, primary care, and pharmacy prescribing data are required to clarify the true incidence of tirzepatide-associated pancreatitis. Incorporating biobank and genetic linkage data may also help identify susceptible individuals and elucidate potential biological mechanisms.

## Conclusions

This retrospective, single-centre audit aimed to assess the occurrence of pancreatitis among inpatients receiving tirzepatide over a 12-month period. Among 222 pancreatitis admissions, four (1.8%) patients were taking tirzepatide; all presented with mild disease, and three had confounding aetiologies such as gallstones or alcohol use. This finding reflects admission prevalence within the audit cohort, indicating that pancreatitis among tirzepatide users was uncommon (1.8%). However, the true drug-related risk cannot be calculated, as overall prescribing data were unavailable and the analysis was limited by its retrospective, single-centre design. Multicentre, prospective studies integrating hospital, primary care, and pharmacy data are needed to establish incidence and clarify causality; by analogy with bariatric surgery, short-term ursodeoxycholic acid prophylaxis could be evaluated as a hypothesis in high-risk tirzepatide users.
